# A 3D Printed Self-Sustainable Cell-Encapsulation Drug Delivery Device for Periocular Transplant-Based Treatment of Retinal Degenerative Diseases

**DOI:** 10.3390/mi11040436

**Published:** 2020-04-21

**Authors:** Hideto Kojima, Bibek Raut, Li-Jiun Chen, Nobuhiro Nagai, Toshiaki Abe, Hirokazu Kaji

**Affiliations:** 1 Department of Finemechanics, Graduate School of Engineering, Tohoku University, 6-6-01 Aramaki, Aoba-ku, Sendai 980-8579, Japan; h.kojima1501@gmail.com (H.K.); bibek.raut03@gmail.com (B.R.); lij.c29@gmail.com (L.-J.C.); 2 Division of Clinical Cell Therapy, United Centers for Advanced Research and Translational Medicine (ART), Tohoku University Graduate School of Medicine, 2-1 Seiryo, Aoba-ku, Sendai 980-8575, Japan; nagai@med.tohoku.ac.jp (N.N.); toshi@oph.med.tohoku.ac.jp (T.A.); 3 Department of Biomedical Engineering, Graduate School of Biomedical Engineering, Tohoku University, 6-6-01 Aramaki, Aoba-ku, Sendai 980-8579, Japan

**Keywords:** retinal degenerative disease, cell-encapsulation device, periocular implant, growth factors, brain-derived neurotrophic factor (BDNF), cell sheet engineering, 3D printing, minimally invasive device

## Abstract

Self-sustainable release of brain-derived neurotrophic factor (BDNF) to the retina using minimally invasive cell-encapsulation devices is a promising approach to treat retinal degenerative diseases (RDD). Herein, we describe such a self-sustainable drug delivery device with human retinal pigment epithelial (ARPE-19) cells (cultured on collagen coated polystyrene (PS) sheets) enclosed inside a 3D printed semi-porous capsule. The capsule was 3D printed with two photo curable polymers: triethylene glycol dimethacrylate (TEGDM) and polyethylene glycol dimethylacrylate (PEGDM). The capsule’s semi-porous membrane (PEGDM) could serve three functions: protecting the cells from body’s immune system by limiting diffusion (5.97 ± 0.11%) of large molecules like immunoglobin G (IgG)(150 kDa); helping the cells to survive inside the capsule by allowing diffusion (43.20 ± 2.16%) of small molecules (40 kDa) like oxygen and necessary nutrients; and helping in the treatment of RDD by allowing diffusion of cell-secreted BDNF to the outside environment. In vitro results showed a continuous BDNF secretion from the device for at least 16 days, demonstrating future potential of the cell-encapsulation device for the treatment of RDD in a minimally invasive and self-sustainable way through a periocular transplant.

## 1. Introduction

Retinal degenerative diseases (RDD), such as age-related macular degeneration (AMD) and retinitis pigmentosa (RP), causes progressive damage to the photoreceptor cells of the retina leading to gradual visual decline [1]. Although no permanent cure or prosthetic exists to date, cell culture and animal experiments done with tropic factors, such as brain-derived neurotrophic factor (BDNF) and ciliary neurotrophic factor (CNTF), have shown that they can revive the damaged photoreceptor cells [2,3,4]. However, their delivery to the retina is very challenging [5,6]. For instance, intravenous injection cannot deliver the required amount of BDNF to the retina because BDNF has a very short half-life in blood (0.92 min) [7], and it is impermeable to the blood-retinal barrier [8]. Likewise, topical installation is equally ineffective due to low permeability through multi-cellular cornea and sclera [9,10]. Moreover, intravitreal injection is highly invasive during long term treatment that requires periodic poking of the eyeball which can risk infection [9]. Although minimally invasive delivery of drugs through the blood-retina barrier using focused ultrasound [11] has been proposed, a minimally invasive way of sustained and localized drug delivery is desirable.

We have previously developed transscleral (periocular) implants as a minimally invasive way to deliver drugs to the retina [12,13,14,15]. These implants are generally placed outside the eyeball (subconjunctival, sub-tendon, peribulbar, posterior juxta-scleral, and retrobulbar spaces) without performing a complicated surgery. Additionally, such implants use a shorter transscleral route that allows relatively high permeability of larger drugs (up to 70 kDa) [16,17]. In addition, we designed these devices with a single sided permeable membrane facing the sclera, which increased the drug delivery efficiency by reducing drug elimination by conjunctival clearance. Although these minimally invasive devices allowed long-term (18 weeks [13]) release of pre-loaded drugs, they had to be replaced once the drug ran out. It was also difficult to pre-determine the exact time for device replacement. Thus, a self-sustainable way of drug delivery is desirable.

**Figure 1:**
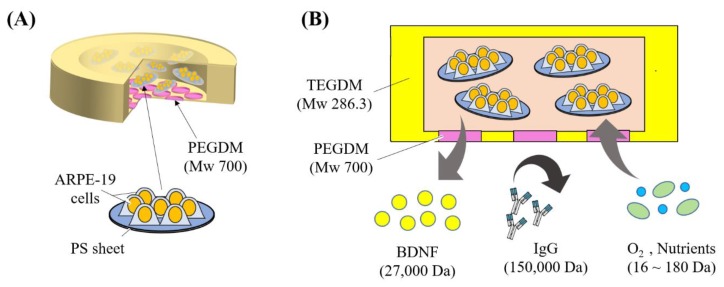
Overview of the cell-encapsulation device. (**A**) A 3D printed capsule with ARPE-19 cells enclosed inside the device. ARPE-19 cells were cultured in polystyrene (PS) sheets. (**B**) Cross-section of device in A. The 3D printed capsule with semi-porous membrane (PEGDM) allowed selective permeability of brain-derived neurotrophic factor (BDNF; 27 kDa), O_2_, and nutrients (16–180 Da) while protecting the cells from the immune response of the body (i.e., immunoglobulin (IgG; 150 kDa)).

A promising way to achieve self-sustainable drug delivery is to replace the drugs in the device with genetically modifiable cells that can continuously secrete trophic factor proteins [18]. In fact, this technique has now gained wide popularity amongst many research groups [5,19]. Herein, we utilized a retinal pigment epithelium (RPE) cell line (ARPE-19; [20]). The RPE cells play an important role in the health of the retina including, but not limited to, the transport of ions, nutrients, and water; absorption of light; and protection against photooxidation [21,22]. RPE cells can also be modified, in principle, to produce almost any trophic factors [18], which makes it highly valuable for treating regenerative diseases. Here, we cultured the ARPE-19 cells on collagen coated polystyrene (PS) sheets and transferred these cell-loaded sheets to a 3D printed capsule (Figure 1). Using the developed cell-encapsulation device, we tested the efficacy of the device in defending the ARPE-19 cells from the body’s immune response (limiting diffusion of molecules bigger than 150 kDa), while simultaneously allowing diffusion of oxygen and nutrients inside the device, and release of BDNF to the outside environment (molecules smaller than 40 kDa). Thus, by utilizing advancement in cell sheet engineering and 3D printing, we developed a self-sustainable cell-encapsulation device that has the potential to be used as a minimally invasive periocular transport for the treatment of retinal diseases.

## 2. Materials and Methods

### 2.1. Materials

The following reagents and all other chemicals used in this study were commercially available and used without further purification: polystyrene (PS, Sigma-Aldrich, St. Louis, MO, USA); polyvinyl alcohol (PVA, Sigma-Aldrich), polyethylene glycol dimethacrylate (PEGDM, MW 750, Sigma-Aldrich); triethylene glycol dimethacrylate (TEGDM, MW 286.3, Sigma-Aldrich); FITC-dextran 40 (FD40, 40kDa, Sigma-Aldrich); FITC-dextran 150 (FD150, 150 kDa, Sigma-Aldrich); FIT7C-IgG (150 kDa, Sigma-Aldrich); Omnirad 819 (formerly Irgacure 819, IGM Resin B.V., Waalwijk, The Netherlands); 2-Isopropylthioxanthone (ITX; photosensitizer, Tokyo Chemical Industry Co., Ltd., Tokyo, Japan). 

### 2.2. Resin Fabrication

Two types of resins were fabricated: PEGDM and TEGDM. PEGDM (Mw 750) was mixed with an equal volume of water (1:1, v/v), while TEGDM (Mw 286.3) was used as it was. Then, 1% photo initiator (Irgacure 819, w/w), and 1% photosensitizer (ITX, w/w) were dissolved into each solution of PEGDM and TEGDM using a magnetic stirrer (4 h, 150 rpm) inside an aluminum wrapped plastic bottle (to protect the solution from light). The resins were filtered with a metallic mesh-type filter (coffee filter with 25 µm holes, Amazon, Tokyo, Japan) to separate aggregates of cured resins from previous printing. 

### 2.3. 3D Printing of Cell Encapsulation Capsule

**Figure A1:**
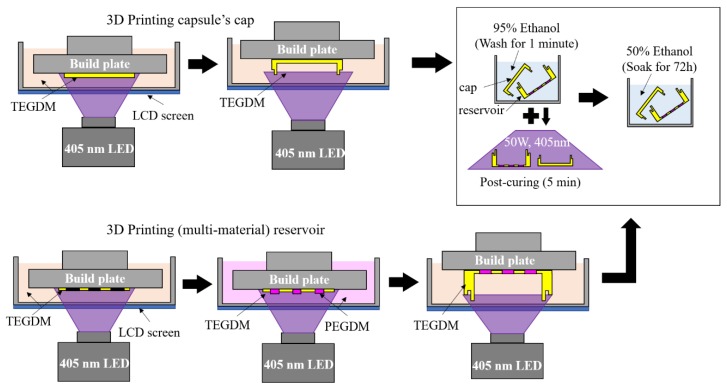
3D printing cell-encapsulation capsule. The cap was printed with TEGDM, whereas the reservoir was printed with both TEGDM and PEGDM. After 3D printing, both parts were washed with 95% ethanol for 1 min, followed by post-curing in UV box (50 W, 405 nm) for 5 min. Finally, 3D printed parts were soaked in 50% ethanol for 72 h to remove photo initiator and photosensitizer and increase biocompatibility of the capsule.

The cell encapsulation capsule consisted of two parts: the cap and the reservoir. Both parts were designed in Solidworks 2018 (Dassault Systèmes, Vélizy-Villacoublay, France) and 3D printed (layer thickness: 50 um) (QiDi tech shadow 5.5 s, QIDI Technology Co., Ltd., Ruian, China) using either TEGDM (cap) or both TEGDM and PEGDM (reservoir) resins (Figure A1). To print the cap, each layer of TEGDM resin was exposed for 8 s (first layer was exposed for 30 s to allow adhesion to the build plate). However, to print the reservoir, a semi-autonomous approach was used. The first layer (exposure time: 30 s) was printed with TEGDM. Then, the printer was paused and the build plate was removed, followed by build plate cleaning with 95% ethanol. TEGDM resin was replaced with PEGDM, and second layer was printed (exposure time: 40 s). The printer was again paused, the build plate removed and washed with ethanol. Then, PEGDM resin was replaced with TEGDM, and the remaining layers were printed normally with TEGDM resin (exposure time: 8 s). After the parts were fully printed, they were washed with 95% ethanol for 1 min and post-cured in a UV box (50 W, 405 nm) for 5 min. Finally, both parts were soaked in 50% ethanol for 72 h to remove the photo initiator and the photosensitizer to improve biocompatibility of the device [23]. A detailed fabrication procedure is shown in Figure A1. 

### 2.4. Cell Culture and Cell Counting

The human retinal pigment epithelial cell line (ARPE-19) was purchased from the American Type Culture Collection (ATCC, Manassas, VA, USA) and routinely sub-cultured (cell seeding density: 40,000 cell/cm^2^). They reached around 70% confluency, in Dulbecco’s Modified Eagle Medium (DMEM) supplemented with 10% fetal bovine serum (FBS, S 1400-500, Biowest, France) and 1% Antibiotic-Antimycotic (100X, Gibco, 15240062) at 37 °C in a humidified condition under 5% CO_2_. Once the cells were confluent, they were detached from the cell culture flask by adding 0.25% (w/v) trypsin and 0.1% (w/v) EDTA and incubating in a cell-culture incubator for 4 min. The detached cells were suspended in a fresh medium and centrifuged at 1500 rpm for 5 min, before removing the medium and resuspending the cells in fresh medium.

To count the total number of cells, the first image was converted to RGB stack and the background was removed and converted to binary image. A 200 µm × 200 µm image area was defined and image processing functions (fill hole, and convert to mask) were used to differentiate the cells from the background. Finally, particle analysis function was used to count the cells.

### 2.5. PS Sheet Fabrication

**Figure A2:**
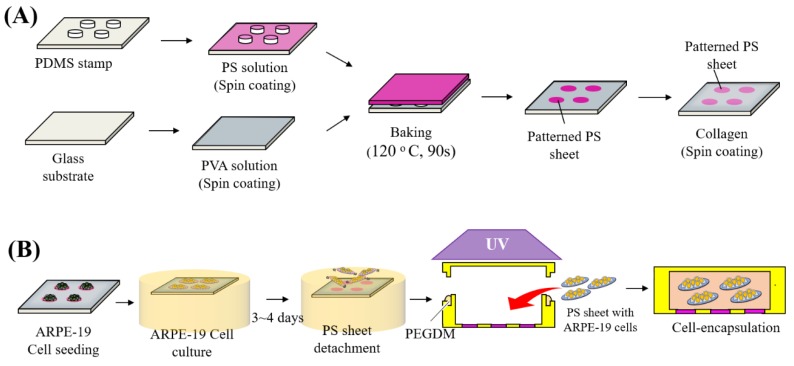
Fabrication of PS sheet, cell culturing, and cell encapsulation. (**A**) PS sheets were patterned in a glass substate coated with PVA. Collagen was spin coated on the PS sheet to culture ARPE-19 cells. (**B**) ARPE-19 cells cultured on the PS sheet could be easily removed once the PVA substrate was dissolved. Cells were then encapsulated inside the capsule by bonding the cap and the reservoir with a UV curable PEGDM resin.

The polystyrene (PS) sheet for cell culture was prepared by a combination of spin-coating and contact printing techniques, as previously described [24]. Briefly, polydimethylsiloxane (PDMS) stamps with convex columns were fabricated by conventional photolithography using SU-8 molds. PS was dissolved in dichloromethane to obtain a 400 mg/mL concentration of PS solution which was spin coated onto the PDMS stamp (4000 rpm, 10 s). Likewise, 10 mg/mL PVA solution was spin coated on a glass substrate (4000 rpm, 40 s). As shown in Figure A2A, the glass plate and the PDMS stamp were pressed against each other with the help of a clip, and baked (120 °C, 90 s). After baking, PS sheet was transformed onto the PVA coated glass plate. The PS patterned glass was then spin coated with 0.5 mg/mL collagen (4000 rpm, 40 s; Cell Matrix^®^ type 1-A collagen, Nitta Gelatin Co., Ltd.). The average diameter and thickness of the PS sheets were 5 mm and 150 nm, respectively.

### 2.6. Cell Encapsulation Inside the 3D Printed Capsule

ARPE-19 cells were seeded onto the collagen coated PS sheet (diameter: 5 mm) with a cell density of 1.0 × 10^5^ cells/mL. Cell were incubated for 2 h to allow adherence to the PS sheets. After that, the glass plate containing the cell seeded PS sheets was gently washed with PBS- to remove excess and unattached cells. Then, cell loaded glass was dipped in a flask containing cell culture medium and incubated in the cell culture incubator. After 3–4 days, cells grew confluent on the PS sheet, and PVA got completely dissolved in the medium. The PS sheets were then removed from the glass substrate by first gently washing with cell culture medium and transferring it to the 3D printed capsule with the help of a tweezer. A total of 12 PS sheets were transferred into each 3D printed reservoir containing 690 µL medium. After transferring the cell containing PS sheets, and filling it with the cell culture medium, both the reservoir and cap were permanently bonded using PEGDM and UV curing for 30 s. The detailed fabrication procedure is shown in Figure A2B.

### 2.7. In-Vitro Diffusion Experiment with Semi-Porous Insert Device

**Figure A3:**
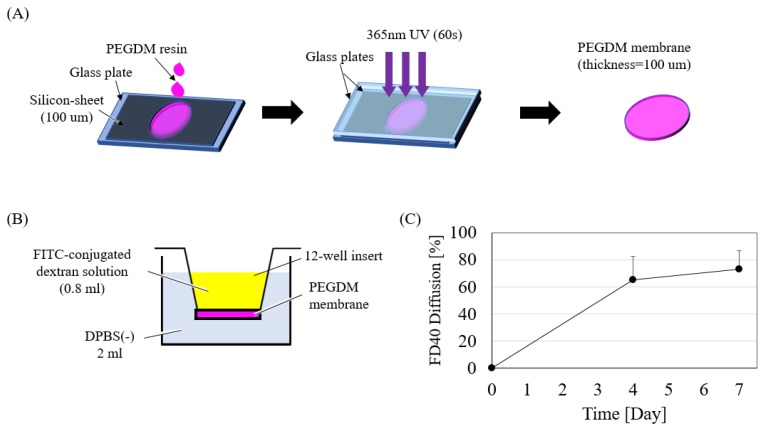
Fabrication of PEGDM membrane and in-vitro diffusion test. (**A**) PEGDM resin sandwiched between glass and a 100 µm thickness silicon-sheet with a round cut was cured using 365 nm UV lamp for 60 s. (**B**) The 12-well transwell insert membrane was replaced with a PEGDM membrane for diffusion test. (**C**) Percentage diffusion of FD40 (n = 3). The error bar represents standard deviation.

The semi-porous membrane was fabricated by curing the PEGDM resin, sandwiched between glass and a 100 µm thickness silicon mold, with a 365 nm UV lamp (Lightningcure LCS, Hamamatsu Photonics, Hamamatsu City, Japan) for 60 s at an intensity of 310 mW/cm^2^ (Figure A3A). The resulting membrane was glued using an acrylate based instant adhesive (Aron Alpha^®^, Tokyo, Japan) to the base of a 12-well insert (Falcon #353180) after removing the existing PET membrane from it. For the diffusion experiment, each insert was loaded with 800 µL FITC-conjugated dextran of either 40 kDa, 150 kDa, or FITC-IgG each of 100 μg/mL concentration, and the well was filled with 2 mL PBS- (Figure A3B). Here, dextran of different molecular weights was used as an evaluation of molecular transport and permeability. The 12-well plate was then put inside a humidified cell culture incubator at 37 °C for 7 days. An end point diffusion measurement was done by taking 200 µL sample from the well and measuring absorption intensity of the sample using a fluorescence plate reader (FluoroscanAscent, Thermo Fisher Scientific, Waltham, MA, USA). Based on a pre-calibrated standard curve, the amount of diffused reagent was determined.

### 2.8. Viability of ARPE-19 Cells Cultured on PS Sheets

ARPE-19 cells cultured on PS sheets were stained using Calcein-AM and Propidium Iodide (PI; Cellstain^®^ Double Staining Kit, Dojin Chemical Lab) at a concentration of 2 µmol/L and 4 µmol/L with PBS-, respectively, following the manufacturer’s staining protocol. Microscopic images were captured using a fluorescent microscope (Olympus, Tokyo, Japan) or a confocal microscope (LSM700, Carl Zeiss MicroImaging, Gina, Germany) on day 0, 9, 15, 22, and 30 and analyzed using the Image-J software (Fiji). The cell viability was measured by first converting the Calcein-AM stained (live cells) images to 8-bit grayscale, then image thresholding function was used to eliminate noise. The percentage of live cell coverage on PS sheets was used to obtain cellular viability.

### 2.9. Quantification of BDNF Release from ARPE-19 Cells

Two separate experiments were done to calculate BDNF expression from ARPE-19 cells. In the first group, BDNF release was measured by taking 100 µL of the medium sample (with replacement) from a fully confluent ARPE-19 monolayer cultured in a 6-well plate (Falcon^®^, #353502), with 3 mL medium inside. In the second group, PS sheets (with confluent ARPE-19 cells) containing capsules were put inside a 6-well plate containing 3 mL cell culture medium. BDNF was measured by taking a 100 µL medium sample from each well. To calculate the amount of BDNF secreted by the cells, 100 µL medium from each sample was measured using the ELISA kit (Rat BDNF ELISA Kit, RayBio). Based on a pre-calibrated absorption curve, the mass of secreted BDNF was determined by multiplying the concentration obtained by ELISA to the amount of medium sampled.

## 3. Results and Discussion

### 3.1. In-Vitro Diffusion Test

**Figure 2:**
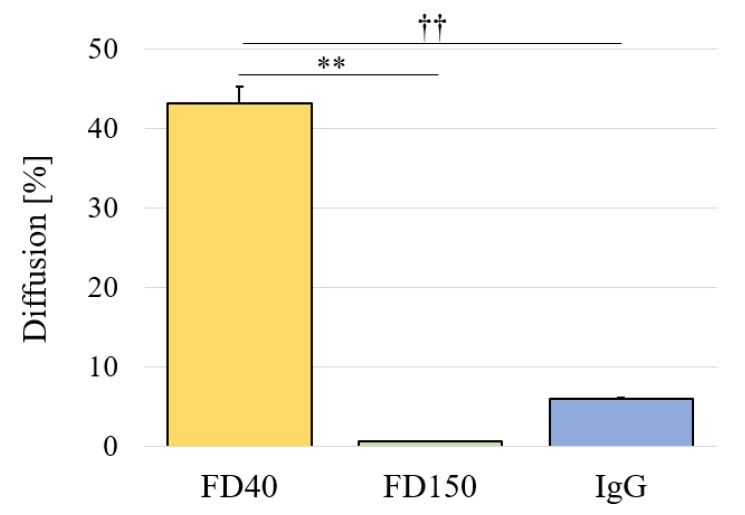
Graph of percentage diffusion of different FITC-conjugated dextran and immunoglobin G (IgG) taken at day 7 (n = 3 for each group). The experiment was conducted in 12-well transwell inserts by replacing the insert’s original PET membrane with a PEGDM membrane (thickness: 100 μm). Error bar indicates standard deviation; ** and †† indicate p < 0.00005 and p < 0.00001 respectively.

The cell-encapsulation device should serve three functions: protection of cells from the body’s immune system; exchange of oxygen and necessary nutrients; and release of BDNF. Molecular weight of nutrients range from 16–180 Da [25], BDNF is 27 kDa [26], and immunoglobulins are larger than 150 kDa [25]. Thus, a semi-porous membrane should be able to allow diffusion of molecules bigger than 27 kDa, but limit diffusion of molecules larger than 150 kDa. As shown in Figure A3B, the in-vitro diffusion test was conducted in a transwell insert by replacing existing PET membranes with 100 µm thickness PEGDM membranes. Figure A3C shows the time course of diffusion of FD40 over day 7. As shown in Figure 2, the PEGDM semi-porous membrane allowed 43% of 40 kDa molecules to pass through, while limiting both 150 kDa and IgG molecules to less than 6% diffusion. Although, FD150 and IgG have the same molecular weight, their diffusion through PEGDA differ significantly. This could be due to the difference in the shape of the molecules (FD150 is a string like molecule, whereas IgG is spherical one). Likewise, the diffusion coefficient for FD40, FD150, and IgG were 4.66 ± 0.13, 0.072 ± 0.001, and 0.63 ± 0.01 × 10^−14^ m^2^/s, respectively, suggesting that the PEGDM membrane is suitable for use in construction of the cell encapsulation device.

### 3.2. High Throughput Fabrication of Cell-Capsule

**Figure 3:**
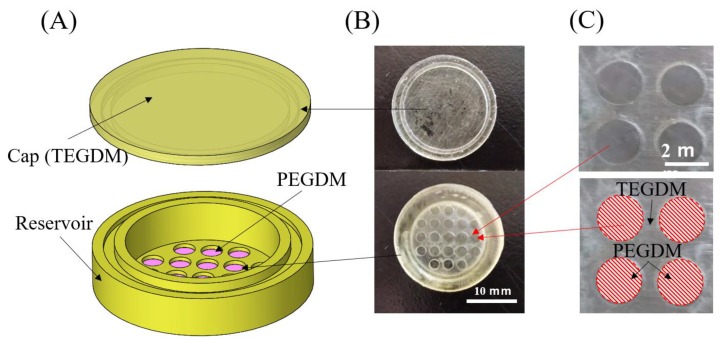
3D printed cell encapsulation capsule. (**A**) 3D exploded view of the CAD model. (**B**) Top view of the actual fabricated device (**C**) Magnified view of the reservoir showing multi-material printing of a semi-porous PEGDM membrane and non-porous TEGDM outer covering.

**Figure A4:**
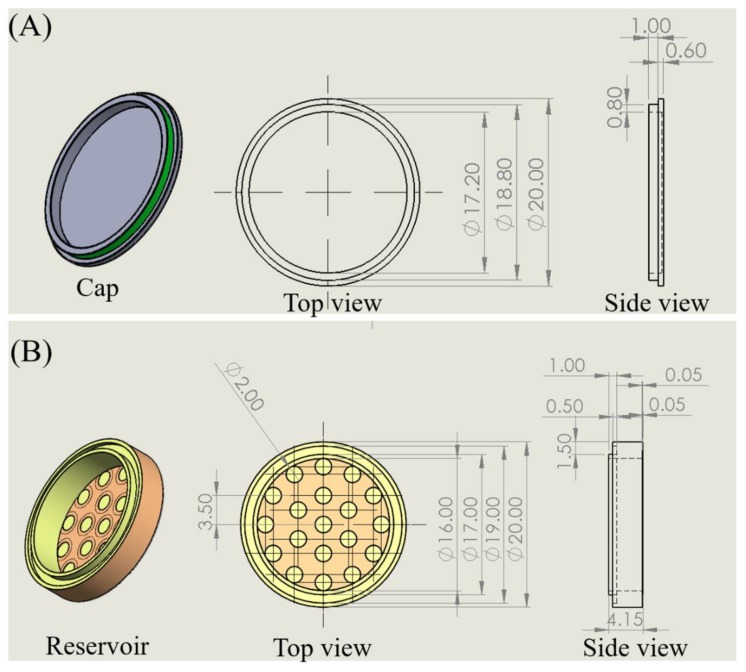
Detail dimension of the cell encapsulation capsule showing the isometric, top, and side view of (**A**) the cap and (**B**) the reservoir. Dimension labeling is in mm.

3D printing is gaining popularity in medical application [27]. Previously, we had utilized mold technique to fabricate drug delivery devices (DDS) [14,15], but it was laborious. Here, with the 3D printing technique, we were able to shorten device prototyping time (on average, mold technique allowed 3–4 devices to be made in 1 h, whereas 20–25 devices could be printed in the same time interval using 3D printing) and cut prototyping time by almost 4–5 times. Thus, utilizing the new 3D printing technique, we manufactured the cell-capsules in two parts: the cap and the reservoir (detailed dimensions are given in Figure A4). As shown in Figure 3, the cap was 3D printed with non-porous TEGDM material, while the reservoir was made by a semi-autonomous multi-material 3D printing technique. Although, there were few multi-material 3D printers on the market, these printers were designed for industrial applications only and are quite expensive. Therefore, we utilized a semi-manual technique whereby we printed a part up to the desired height, then paused the printer and replaced the material with another one (Figure A1). This process was repeated until we achieved a desired multi-material capsule. The major advantage of 3D printing is that it allows fast prototyping of complex 3D geometry that is not possible with conventional manufacturing techniques like milling or laser cutting. Although we used a simple cylindrical box design for in-vitro tests, it should be possible to 3D print a more realistic periocular transplant device with a curved plate geometry that fits the curvature of the eye. 

### 3.3. Cell Viability of ARPE-19 Cells Cultured in Polystyrene (PS) Sheet

**Figure 4:**
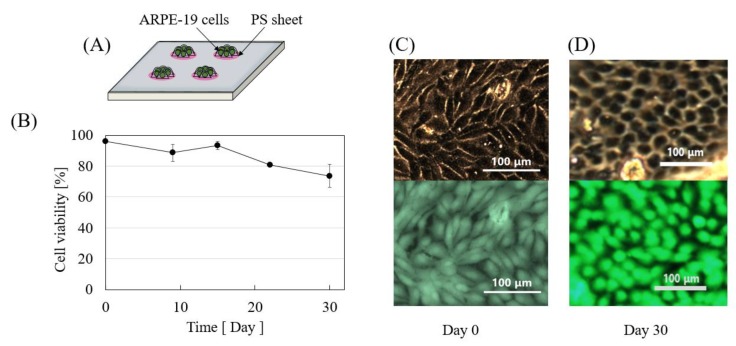
Cellular viability of ARPE-19 cells in collagen coated PS sheets. (**A**) Cells were cultured on collagen coated 5 mm diameter PS sheets. (**B**) ARPE-19 cells maintain over 75% cell viability for at least 30 days on the PS sheet (n = 3). Morphology of confluent monolayer cells are similar for both (**C**) day 0 and (**D**) day 30.

**Figure 5:**
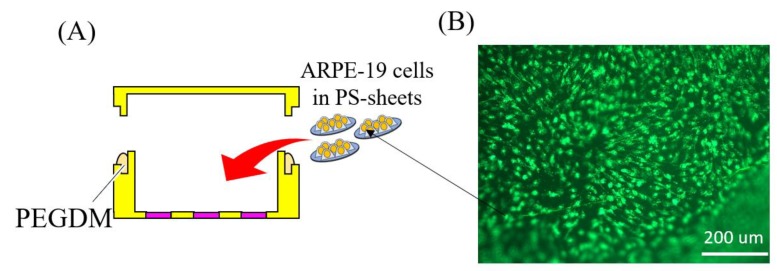
Cellular viability of ARPE-19 cells inside cell encapsulation capsule. (**A**) Conceptual image of the cell cultured PS sheets encapsulation process. (**B**) Image of ARPE-cells in 5 mm PS sheets on day 3.

Since a self-sustainable cell-encapsulation device needs to function for several months to years, viable cells at the optimal state are paramount. Here, we cultured ARPE-19 cell on a collagen coated PS sheet (Figure 4A). Cell sheet technology has been widely utilized for cellular transplant in tissue engineering and regenerative medicine applications [24,28]. Utilizing this technique, we successfully cultured ARPE-19 cells on collagen coated PS sheets. As shown in Figure 4B, cells cultured on PS sheets maintained more than 75% viability for at least 30 days. ARPE-19 cells form monolayers with a polygonal shape [20]. As can be seen in Figure 4C,D, ARPE-19 grew confluent and maintained a polygonal shape on day 30. Likewise, as shown in Figure 5A, ARPE-19 containing PS sheets were enclosed inside the cell encapsulation capsule. PS sheets taken out from the capsule on day 3 also show good cell viability (Figure 4B). A total of 12 PS sheets (5 mm diameter each) was enclosed inside a capsule diameter (20 mm) × height (4.15 mm), however the sheet density inside the capsule can be increased by utilizing micro sheets [28].

### 3.4. BDNF Release from the Cell-Encapsulated Device

**Figure 6:**
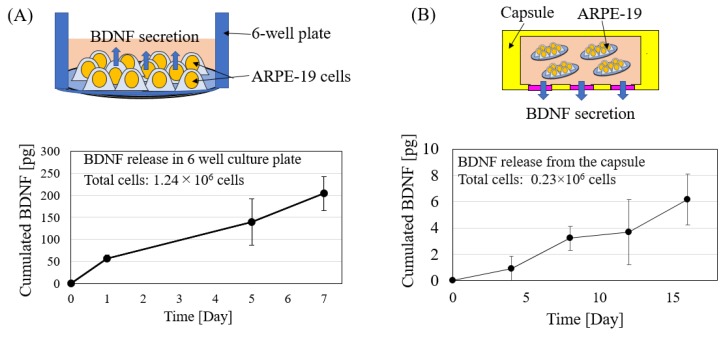
Total amount of BDNF collected from ARPE-19 cultured in (**A**) 6-well plate and (**B**) cell-encapsulated capsule. Image inserts show illustration of the experiment conducted. The error bars indicate standard deviation (n = 3 samples for each group).

Two tests were conducted to evaluate BDNF secretion from ARPE-19 cells. The first test (control group) consisted of a confluent monolayer of ARPE-19 cells grown in a 6-well culture plate, while the second one (experimental group) contained confluent cells grown on 12 PS sheets that were embedded inside the capsule. Figure 6 shows cumulative BDNF release in both groups. As expected, BDNF secretion increased monotonically in the control group (6-well plate) and the experimental group (capsule). In the experimental group in particular, BDNF secretion was observed for 16 days. However, total BDNF release from the capsule was around 3.5 times (normalized to total cell count and percentage of BDNF diffusion through the capsule) lower than the one measured in the 6-well culture plate (in reference to data from day 7). One possible reason that a low BDNF was observed in the experimental sample compared to the control could be because BDNF gradient inside the capsule and outside the capsule was much smaller initially. In addition, for actual therapeutic effect on retinal degenerative diseases, administration of several ng/mL or more is necessary [2,3]. Although, we utilized a non-engineered ARPE-19 cell line as a proof-of-concept study, it is possible to increase BDNF secretion by genetically engineering ARPE-19 cells. In fact, such transgenic technology already exists and several groups have reported secretion of several neurotrophic factors, including BDNF in the order of ng/mL [29,30,31,32]. Utilizing such genetically engineered ARPE cells, it should be possible to improve the performance of the device further.

## 4. Conclusions and Future Work

We built a cell encapsulation device by 3D printing a semi-porous capsule, with ARPE-19 cells grown on collagen coated PS sheets enclosed inside. Prior to 3D printing the semi-porous capsule, we conducted diffusion experiments and observed that the photocurable PEGDM membrane was capable of limiting diffusion of large molecules (150 kDa) to protect the cells from the body’s immune factor molecules, while simultaneously allowing passage of smaller molecules (40 kDa) like oxygen and nutrients that are essential for cell survivability, and BDNF molecules that are needed for the treatment of retinal diseases. Further, ARPE-19 cells cultured on collagen coated PS sheets were able to maintain 75% confluency for at least 30 days outside the device, and were able to secrete BDNF for at least 16 days while inside the device. The proposed cell-encapsulation device has the potential to be used as a self-sustainable way to deliver BDNF to the retina through minimally invasive periocular transplant. In the future, we plan to increase BDNF release further by utilizing genetically modified ARPE-19 cells and testing the therapeutic potential on an animal model.
